# Plasmonic-Layered InAs/InGaAs Quantum-Dots-in-a-Well Pixel Detector for Spectral-Shaping and Photocurrent Enhancement

**DOI:** 10.3390/nano10091827

**Published:** 2020-09-13

**Authors:** Jehwan Hwang, Zahyun Ku, Jiyeon Jeon, Yeongho Kim, Jun Oh Kim, Deok-Kee Kim, Augustine Urbas, Eun Kyu Kim, Sang Jun Lee

**Affiliations:** 1 Interdisciplinary Materials Measurement Institute, Korea Research Institute of Standards and Science, Daejeon 34113, Korea; wowhwang87@gmail.com (J.H.); jeony210@gmail.com (J.J.); ykim172@kriss.re.kr (Y.K.); 2 Department of Physics and Research Institute for Convergence of Basic Sciences, Hanyang University, Seoul 04763, Korea; 3 Materials and Manufacturing Directorate, Air Force Research Laboratory, WPAFB, OH 45433, USA; zahyun.ku.1.ctr@us.af.mil (Z.K.); augustine.urbas@us.af.mil (A.U.); 4 Department of Electronic Engineering, Sejong University, Seoul 05006, Korea; deokkeekim@sejong.ac.kr; 5 Advanced Instrumentation Institute, Korea Research Institute of Standards and Science, Daejeon 34113, Korea; jokim@kriss.re.kr

**Keywords:** spectral imaging, plasmonic resonance, metal hole array, quantum dots-in-a-well, electromagnetic simulation

## Abstract

The algorithmic spectrometry as an alternative to traditional approaches has the potential to become the next generation of infrared (IR) spectral sensing technology, which is free of physical optical filters, and only a very small number of data are required from the IR detector. A key requirement is that the detector spectral responses must be engineered to create an optimal basis that efficiently synthesizes spectral information. Light manipulation through metal perforated with a two-dimensional square array of subwavelength holes provides remarkable opportunities to harness the detector response in a way that is incorporated into the detector. Instead of previous experimental efforts mainly focusing on the change over the resonance wavelength by tuning the geometrical parameters of the plasmonic layer, we experimentally and numerically demonstrate the capability for the control over the shape of bias-tunable response spectra using a fixed plasmonic structure as well as the detector sensitivity improvement, which is enabled by the anisotropic dielectric constants of the quantum dots-in-a-well (DWELL) absorber and the presence of electric field along the growth direction. Our work will pave the way for the development of an intelligent IR detector, which is capable of direct viewing of spectral information without utilizing any intervening the spectral filters.

## 1. Introduction

Spectral imaging, also called imaging spectrometry [1,2], is a technology that acquires image information of space and each spectral information at a given scene at the same time and implements it into a spectroscopic imaging data cube. Differently from infrared (IR) imaging acquiring data over the entire wavelength response range of the IR imager, IR spectral imaging uses a spectroscopic system to obtain a series of consecutive spectra for each image pixel. A spatially and spectral-resolved image of the object is analyzed to identify the materials compositions of the object in a scene, and to classify materials that are undistinguishable with conventional methods [3,4,5]. Traditional IR spectral imaging acquires spectral information by placing an optical system such as dispersive or filter-based spectrometers in front of an IR detector [6,7,8], however there are obstacles to be overcome such as cost, complexity, calibration, and real-time detection. The algorithmic spectrometry [9,10,11,12,13,14,15,16] as an alternative to traditional approaches has been recently developed, and its concept is to reconstruct the spectrum of an unidentified object at a wavelength of interest without utilizing any intervening spectral filters, which is enabled by finding the optimal set of weights by the projection algorithm [17,18,19]. A set of weights is used to synthesize the arbitrary bandpass filter, which optimally approximates a desired spectral filter shape with a specified center wavelength and bandwidth [20,21]. More importantly, an essential prerequisite to fully benefit from the algorithmic spectrometer for the object detection largely relies on the ability to easily modify the IR detector’s responsivity, e.g., peak wavelength, bandwidth, and spectral shape. For this purpose, the physical phenomenon of plasmonic resonance in a metal film perforated with a two-dimensional subwavelength hole array has been the focus of significant interest recently [22,23,24], because the plasmonic resonance wavelength is simply related to the structural periodicity as well as the detector sensitivity can be improved by integrating the plasmonic resonance layer onto IR detector, resulting from enhancing the coupling to the active region through local field engineering at the plasmonic resonance wavelengths. It is worth pointing out that this perforated metal film structure unconditionally needs to play with the quantum dots (QDs)-based IR detectors [25,26,27,28,29,30,31,32,33,34,35,36,37] for spectral-shaping and photocurrent enhancement, which is caused by the anisotropic dielectric constants of the active layer, specifically, the different absorption efficiencies of active layer into *x*- or *y*-direction (lying in the plane of perforated metal film) and *z*-direction (along the growth direction) [38,39,40,41,42,43,44,45]. It is also worth referring to research on a hybrid device consisting of artificial structure and thermal detector (that can operate at or even above room temperature without cryogenic cooling), which has seen impressive growth, exhibiting the spectral selectivity at the desired wavelength [46,47,48,49,50,51,52,53,54,55,56,57,58]. Over the past years, there have been experimental efforts to demonstrate the change of peak wavelength photoresponse at a fixed bias by structural tuning of plasmonic structure (e.g., periodicity or aperture size) [38,43,44,45]. In contrast, we focus in this letter on control over the shape of bias-dependent wavelength-tunable response spectra using a fixed plasmonic structure (demonstrating the possibility to modify the IR detector’s response spectra as desired and to increase the number of different IR detector’s responsivities in order to efficiently improve the outcome from the projection step), and at the same time, show the detector responsivity enhancement at the plasmonic resonance wavelength.

Three to five self-assembled quantum dots (QDs) are a promising candidate for light-absorbing materials used for the so-called fourth generation IR imagers [8]. The absorption band of InAs QDs is varied from mid–wave IR (MWIR, 3–5 µm) to long–wave IR (LWIR, 8–14 µm) by manipulating the dot geometries and strain fields [27,29]. Strong normal incidence photodetection is possible through intersub-band transitions in QDs due to the three-dimensional confinement of the charge carriers [30]. Reduced electron–phonon scattering in QDs increases the lifetime of photo-excited electrons, leading to high photoconductive gain and high responsivity of QD-based IR photodetectors (QDIPs). Additionally, high temperature operation of QDIPs is feasible owing to the low dark current resulting from a large thermal activation energy, defined as the energy necessary for a bound electron to be excited out of the QD [31,37]. A novel type of intersub-band QDIP using a quantum dots-in-a-well (DWELL) heterostructure has been developed to combine the advantages of quantum wells (QWs) with the advantages of QDs, for instance control over the operating wavelength and normal incidence, respectively [25,26,27,28,32,33,34,35,36,37]. Furthermore, multi-stacking DWELL layers with a uniform dot size and a high dot density is required to improve the absorption quantum efficiency, thereby enabling the realization of high-performance DWELL-based focal plane array (FPA) device (high spatial uniformity and high operability). However, it is challenging to grow such DWELL heterostructures due to strain accumulation and the generation of crystalline defects. Another feasible way to improve the quantum efficiency is to incorporate plasmonic nanostructures into a DWELL-FPA for light absorption enhancement in a given volume of QDs, as described above. Besides enhanced light absorption, plasmon-enhanced DWELL detectors provide many advantages such as wavelength controllability, low 1/*f* noise performance, and high thermal sensitivity [44,45,59,60].

## 2. Bias-Tunable Quantum Dots-in-a-Well Pixel Detector

**Figure 1:**
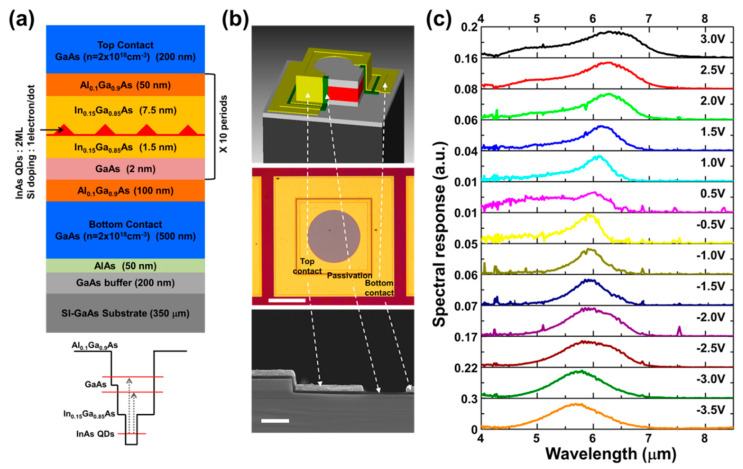
(**a**) Schematic structure (upper) and conduction band diagram (lower) of bias-dependent dots-in-a-well (DWELL) heterostructure; (**b**) illustration (upper), optical microscope image (middle), and cross-section view scanning electron microscope (SEM) image (bottom) of the reference DWELL-based single-pixel IR detector. Scale bars are 200 µm (middle) and 2 µm (lower); (**c**) bias-dependent spectral responses of DWELL pixel detector at 77 K.

The quantum dots-in-a-well (DWELL) heterostructure was grown on (001) GaAs substrate by molecular beam epitaxy (MBE) system. As shown in Figure 1a, the DWELL-based single-pixel IR detector (DWELL pixel) consists of a 200 nm-thick GaAs buffer layer, a 50 nm-thick AlAs etch stop layer, a 500 nm-thick n-type GaAs bottom contact layer, active quantum dot (QD) absorption layers, and a 200 nm-thick n-type GaAs top contact layer. The top and bottom contact layers were doped with silicon with a doping concentration of n = 2 × 10^18^ cm^–3^. The active layer consisting of 10 periods of the DWELL structure was sandwiched between the top and bottom contact layers, separated by 50 nm Al_0.1_Ga_0.9_As barriers. The InAs QDs with a nominal thickness of 2 monolayers were embedded in an asymmetric quantum well (QW) structure formed by 50 nm Al_0.1_Ga_0.9_As/7.5 nm In_0.15_Ga_0.85_As/1.5 nm In_0.15_Ga_0.85_As/2 nm GaAs/Al_0.1_Ga_0.9_As layers. The InAs QDs were doped with silicon of a doping concentration of ~1 electron/dot for high photon absorption. 

As shown in the upper and middle panels of Figure 1b, the reference DWELL pixel had a mesa structure with a circular aperture of a diameter of 300 µm. Each mesa was isolated by inductively coupled plasma (ICP) etch with BCl_3_ chemistry, followed by a wet etch with a citric acid-based solution. After the mesa formation, a 200 nm-thick Si_3_N_4_ as a surface passivation layer was deposited using plasma enhanced chemical vapor deposition (PECVD). The ohmic contact metals of Ge (26 nm)/Au (54 nm)/Ni (15 nm)/Au (350 nm) were deposited on the top and bottom contact layers using an e-beam evaporator and annealed at 380 °C for 1 min. Note that the mesa sidewalls were covered with the top contact metals to prevent unwanted light from entering the sidewalls of the DWELL pixel, as clearly seen in the scanning electron microscope (SEM) image of lower panel of Figure 1b. The bias-dependent spectral responses of the DWELL pixel were measured at 77 K for normal incidence using a Fourier transform infrared (FTIR) spectrometer (Nicolet 5700) and a low-noise current amplifier (Keithley 428). Figure 1c shows that the peak position of the DWELL pixel spectral response is red-shifted continuously from 5.70 µm to 6.28 µm with increasing the bias voltage from −3.5 V to +3.0 V. This bias tunability of the spectral response is due to the enhanced quantum-confined Stark effect in the asymmetric DWELL structure [61,62]. The intersub-band spacing between the ground state of the QD and the excited state of the InGaAs QW decreases with increasing the applied electric field, as indicated in Figure 1a.

## 3. Aperture Shape Variation Effect in the Fabricated Plasmonic Layer on DWELL Pixel Detector 

**Figure 2:**
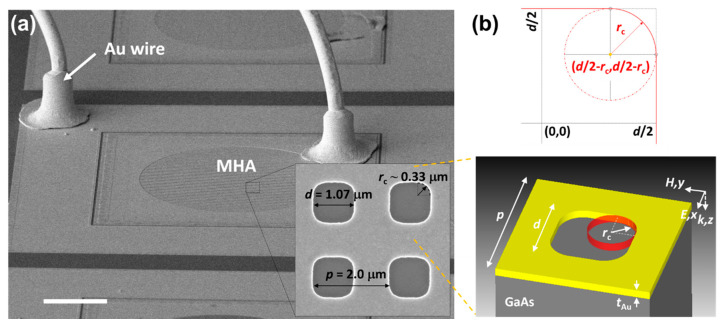
(**a**) 20° tilted SEM image of metal hole array (MHA)-layered DWELL pixel detector. Inset shows the magnified square holes with rounded corners: *p* = 2.0 µm, *d* = 1.07 µm, and *r*_c_ = ~0.33 µm. Scale bar is 100 µm; (**b**) schematic illustration and geometrical parameters of MHA/GaAs unit cell with *r*_c_. Here, two extreme cases for the corner-shape radius *r*_c_ are *r*_c_ = 0 µm for a square-shaped hole and *r*_c_ ~ 0.54 µm (*r*_c_/*d* = 0.5) for a circle-shaped hole. In addition, the configuration of polarization (*E*//*x*) and propagation (*k*//*z*) are depicted.

Prior to the integration of a subwavelength metal hole array (MHA) with DWELL pixel, we designed the MHA to place the MHA-DWELL response peak near the bare DWELL response peak at +3.0 V (~6.3 µm, Figure 1c) using the 3D full field electromagnetic simulation (CST Microwave studio [63]) based on a finite integration technique (details will be provided in the following section). The geometrical parameters of MHA were found to be 2.0 µm for the grating period (*p*), 1.0 µm for a side length of square hole (*d*), and 0.1 µm for the Au thickness (*t*_Au_). The designed MHA was fabricated on the aperture of the reference DWELL pixel using a conventional photolithography, e-beam evaporation and lift-off process. Note that the hole-shape discrepancy between the designed and fabricated MHAs is clearly seen in the SEM image, as shown in Figure 2a. The geometrical parameters of the fabricated MHA were found to be *p* = 2.0 µm, *d* = 1.07 µm, *r*_c_ (a corner-shape radius) = ~0.33 µm. All four corners were rounded, which is probably due to imperfections in the fabrication (the optical diffraction limit during the photolithography process [64]). In order to investigate the effect of rounded corners in the MHA layer on the surface plasmon resonance (SPR), we gradually vary the side length *d* and corner-shape radius *r*_c_ and numerically calculate the transmission of MHA on GaAs substrate. In the simulations, a single unit cell, as illustrated in the lower panel of Figure 2b, was simulated. The refractive index of the substrate material (GaAs) was taken as *n*_GaAs_ = 3.4 and the Drude model was used for Au dielectric function [65], with plasma frequency of *ω*_p_ = 1.38 × 10^16^ Hz and collision frequency of *ω*_c_ = 5.71 × 10^13^ Hz.

**Figure 3:**
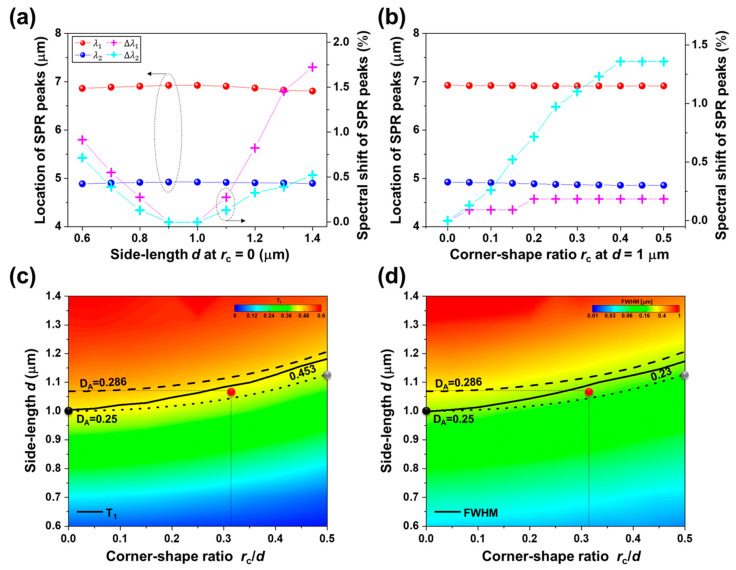
The location and spectral shift of peak transmission at the first- and second-order SPR modes for MHA/GaAs structure as a function of (**a**) the side length *d* with a fixed *r*_c_ at 0; (**b**) the ratio of corner-shape radius to the side length, *r*_c_/*d* with *d* = 1 µm. The first- and second-order SPR peak wavelengths (*λ*_1_ and *λ*_2_) and the spectral shift of SPR-transmission peaks (Δ*λ*_1_ and Δ*λ*_2_) are represented by the red, blue, magenta, and cyan symbols, respectively. The colormaps of (**c**) transmission peak intensity; (**d**) the bandwidth at 50% of the peak transmission (FWHM) at *λ*_1_ = 6.93 µm as a function of *d* (0.6 µm ≤ *d* ≤ 1.4 µm) and *r*_c_/*d* (0 ≤ *r*_c_⁄*d* ≤ 0.5).

Figure 3a shows the simulation results for the SPR peak wavelengths (corresponding to the first- and second-order SPR modes at the interface between MHA layer and GaAs substrate) as a function of side length *d* with a fixed *r*_c_ at 0 µm (i.e., the hole is square-shaped) in addition to the spectral shift of SPR wavelengths (relative to the side length of 1.0 μm, i.e., *λ*_1_ = 6.93 µm and *λ*_2_ = 4.92 µm) as the side length *d* changes. As *d* changes from 1.0 µm, the first- and second-order SPR wavelengths (*λ*_1_, *λ*_2_) tend to blue-shift; specifically, Δ*λ*_1_ = ~63 nm (−0.91%) and Δ*λ*_2_ = ~35 nm (−0.72%) are obtained when *d* = 0.6 µm (Δ*λ*_1_ = ~119 nm (−1.72%) and Δ*λ*_2_ = ~26 nm (−0.52%) when *d* = 1.4 µm). The location and spectral shift of SPR peaks due to varying the corner-shape ratio (*r*_c_/*d* when *d* = 1 µm) of the apertures in the MHA layer from 0 (square-shaped hole) to 0.5 (circle-shaped hole) are also shown in Figure 3b. The *λ*_1_ and *λ*_2_ are blue-shifted by −0.18% (~13 nm) and −1.36% (~67 nm) for increased *r*_c_/*d* from 0 to 0.5, respectively. Despite the variations in the side length and the corner-shape ratio, the SPR peak wavelengths of the *λ*_1_ and *λ*_2_ are nearly kept constant within a few percent, max (|Δ*λ*_1_|) = 1.72%, max (|Δ*λ*_2_|) = 0.72% for error in the side length *d*, and max (|Δ*λ*_1_|) = 0.18%, max (|Δ*λ*_2_|) = 1.36% for error in the corner-shape ratio *r*_c_/*d* ( *r*_c_/*d* in the unit of μm can be considered as the corner-shape radius *r*_c_ because *d* = 1 µm). Thus, the position of SPR transmission peaks of the fabricated MHA layer on DWELL pixel detector could be anticipated to spectrally shift less than ~19 nm (~6 nm) due to 70 nm error in the side-length (i.e., *d* = 1.07 µm) and ~12 nm (~54 nm) resulting from 0.33 µm error in the corner-shape radius (i.e., *r*_c_ = 0.33 µm) for the first (second)-order SPR as compared with the designed MHA layer (*d* = 1.0 µm and *r*_c_ = 0 µm). The SPR excitation wavelength is dominantly determined by the periodicity of the MHA, and also, it is slightly affected by the SPR-scattering-induced phase shift, i.e., depending on the hole geometry [66,67,68,69,70]. 

The first-order SPR peak transmission (T_1_) and the bandwidth at 50% of T_1_ (FWHM) were observed in the range of the corner-shape ratio *r*_c_/*d* from 0 to 0.5 and the side-length *d* from 0.6 µm to 1.4 µm, which are presented in Figure 3c,d using the colormaps to clearly show the dependence of hole geometry due to the imperfections in the fabrication. The ratio of hole area (*A*) to the unit area (*p*^2^ = 2 µm × 2 µm = 4 µm^2^) is referred to as the hole-area density (*D*_A_ = *A*/*p*^2^). The black and gray symbols in Figure 3c indicate (*r*_c_/*d*, *d*) the square- and circle-holes arrays with *D*_A_ = 0.25, specifically (0, 1 µm) and (0.5, ~1.13 µm), respectively. The solid line represents the curve along which the function T_1_ of *r*_c_/*d* and *d* has T_1_ (0, 1 µm) = 0.453, and the *r*_c_ and *d* values producing the same *D*_A_ are indicated as dotted contour lines (*D*_A_ = 0.25 for *r*_c_/*d* = 0 and *d* = 1.0 μm; *D*_A_ = 0.286 for *r*_c_/*d* = 0 and *d* = 1.07 µm). The difference in the first-order SPR transmission peak positions of (0, 1 µm) and (0.5, ~1.13 µm) is ~6.24 nm, and the transmission intensity and FWHM of (0, 1 µm) are found to be 6% higher and 43 nm wider than (0.5, ~1.13 µm). As compared to the designed MHA layer (*D*_A_ = 0.25, black symbol), the hole-area density *D*_A_ of the fabricated MHA layer on DWELL pixel detector (*D*_A_ = ~0.26, red symbol) is ~4% higher due to increased *d* and *r*_c_ by 70 nm and 0.33 µm, respectively; in addition, the first-order SPR transmission intensity of fabricated MHA is slightly decreased by 0.006 (~1.3%), and the FWHM is ~9.1% narrower, which may come from the change in the effective dielectric property in the Au film perforated with subwavelength holes [66,67,68,69,70]. The simulation results in Figure 3 clearly show that the fabrication-induced hole-shape variation in the MHA has little impact on the SPR characteristics, e.g., *λ*_1_ (spectral position of SPR peak), the bandwidth, and T_1_ (SPR-enhanced transmission). The SPR-related simulations (associated with the fabrication-induced imperfections) drawn here will be of great importance in the next section, because they are closely correlated to modifying the spectral selectivity and to enhancing the coupling to the active layer of DWELL pixel detector.

## 4. Underlying Mechanism of Plasmonic Layered DWELL Pixel Detector

**Figure 4:**
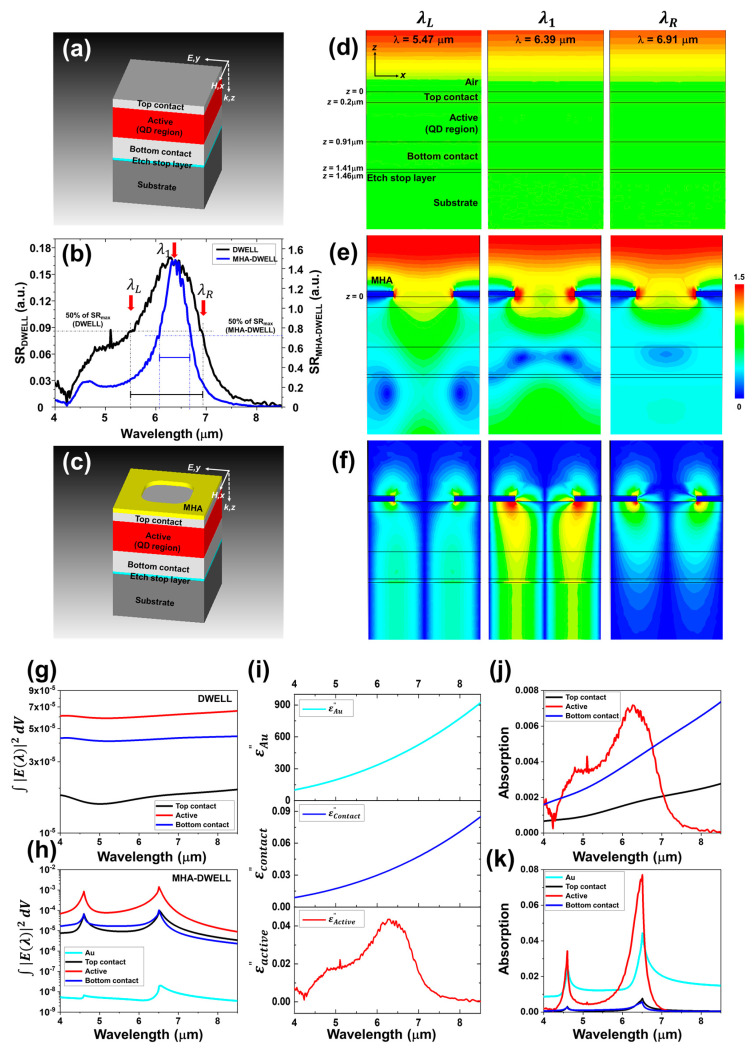
(**a**,**c**) The schematic illustrations of the simplified unit cells of DWELL and MHA-DWELL pixel detectors; (**b**) measured spectral response of DWELL and MHA-DWELL pixel detectors at a bias of +3.0 V and a temperature of 77 K. The simulated *x* component-electric field |*E*_x_| for DWELL and |*E*_x_| and |*E*_z_| for MHA-DWELL at the wavelengths of *λ*_L_ (5.47 µm), *λ*_1_ (6.39 µm), *λ*_R_ (6.91 µm) in a *xz*-plane through the center of unit cell; (**d**) |*E*_x_ (*x*,0,*z*)| for DWELL; (**e**) |*E*_x_ (*x*,0,*z*)|; (**f**) |*E*_z_ (*x*,0,*z*)| for MHA-DWELL. The volume integral of total electric field intensity for all layers constituting (**g**) DWELL; (**h**) MHA-DWELL; (**i**) the imaginary part of dielectric constant in the contact layers (top and bottom), Au, and the active layer. The total absorption of each layer in (**j**) DWELL; (**k**) MHA-DWELL, which are easily calculated by multiplying the layer–volume integral of electric field intensity by ($2\pi C_{0}\cdot \varepsilon_{i}^{\prime \prime }\left(\lambda \right)/\lambda$) (for i-layer, $A_{i}\left(\lambda \right)=\left(2\pi c_{0}\cdot \varepsilon_{i}^{\prime \prime }\left(\lambda \right)/\lambda \right)\times \int {\left|E_{x}\left(\lambda \right)\right|}^{2}dV$ for DWELL and $A_{i}\left(\lambda \right)=\left(2\pi c_{0}\cdot \varepsilon_{i}^{\prime \prime }\left(\lambda \right)/\lambda \right)\times \int ({\left|E_{x}\left(\lambda \right)\right|}^{2}+\eta {\left|E_{z}\left(\lambda \right)\right|}^{2})dV$ for MHA-DWELL). Note that *η* = 7 is applied for the active layer of MHA-DWELL to bring the characteristic of quantum dot (QD)-shape-dependent sensitivity, i.e., the electric field along the growth direction is dominantly interacting with the QDs.

Figure 4b shows the measured spectral response of the DWELL pixel (without MHA layer, referring to ‘DWELL’) and MHA-layered DWELL pixel (with MHA layer, referring to ‘MHA-DWELL’) at a bias of +3.0 V and a temperature of 77 K. The bandwidth at 50% of the peak DWELL response is found to be ~1440 nm, i.e., |*λ*_L_ − *λ*_R_|, where *λ*_L_ = ~5.47 µm and *λ*_R_ = ~6.91 µm at 50% of the peak DWELL response intensity of ~0.085 (the peak DWELL response was detected at ~6.29 µm). The peak MHA-DWELL response was observed at the wavelength of *λ*_1_ ~ 6.39 µm and FWHM was found to be ~610 nm, which is attributed to the fourfold degenerate first-order SPR mode (note that unpolarized FTIR beam was incident to measure the spectral responses of DWELL and MHA-DWELL). In order to investigate the effect of MHA layer (SPR) to the bandwidth (FWHM) reduction, the electric field distributions in *x* (parallel to the incident polarization) and *z* (along the MBE growth direction) directions were simulated at *λ*_L_ (5.47 µm), *λ*_1_ (6.39 µm), and *λ*_R_ (6.91 µm) when an *x*-polarized plane wave is normally incident to the apertures of the DWELL and the MHA-DWELL. The schematic illustrations of the simplified unit cells of DWELL and MHA-DWELL are shown in Figure 4a,c, which were used in the simulation. The DWELL and MHA-DWELL consist of the multiple layers for top contact (0.2 µm), active (0.71 µm), bottom contact (0.5 µm), etch stop (0.05 µm) as well as MHA layer (*p* = 2.0 µm, *d* = 1.07 µm, *r*_c_ = 0.33 µm, *t*_Au_ = 0.105 µm). Figure 4e,f show the simulated *x* and *z* component-electric field (|*E*_x_| and |*E*_z_|) distributions for MHA-DWELL in a *xz*-plane through the center of unit cell. We also present the simulated |*E*_x_| distribution of DWELL to clearly see the difference due to the presence of the MHA layer (SPR effect), as plotted in Figure 4d. Note that |*E*_y_| ≈ 0 and |*E*_z_| ≈ 0 for DWELL and |*E*_y_| ≈ 0 for MHA-DWELL. For DWELL, we can observe the uniformly distributed |*E*_x_| over the whole active area (i.e., *E*_x_ (*x*, *y*, *z*) = *E*_x_ (*z*), where 0.2 µm ≤ *z* ≤ 0.91 µm) and propagating *E*_x_ along *z*-direction. Contrary to the DWELL, when the MHA (integrated with DWELL) interacts with the incident EM wave with a wavelength of *λ*_L_, *λ*_1_, and *λ*_R_, the strong |*E*_x_| and |*E*_z_| are observed at the edges of aperture in the MHA, as shown in Figure 4e,f. The highest value of |*E*_k_ (*λ*)| at the hole edges in the wavelength range of interest is found at the wavelength of *λ*_1_ (|*E*_k_ (*λ*_1_)| > |*E*_k_ (*λ*_R_)| > |*E_k_* (*λ*_L_)|, where *k* = *x* or *z*), which is ascribed to the electric current on the MHA generated by the first-order SPR. In the following discussion, the excited electric field (|*E*_z_|) due to the SPR in the MHA-DWELL will be greatly important role to enhance the DWELL response intensity (photocurrent) in addition to changing the bandwidth (FWHM) of photocurrent spectral response of DWELL. 

The total absorption in each layer of the considered DWELL (or MHA-DWELL) can be calculated using Equation (1), where *i*’s are top / bottom contact layers, active layer, and MHA; *c*_0_ is the speed of light; $\varepsilon_{i}^{\prime \prime }\left(\lambda \right)$ is the imaginary part of dielectric constant; |*E_i_*_,x_(*λ*)|^2^, |*E_i_*_,y_(*λ*)|^2^ and |*E_i_*_,z_(*λ*)|^2^ are the *x*, *y*, and *z* components of the electric field intensity over *i*-layer; *η* ~ 7 is used to account for anisotropic dielectric constant in the active layer.

(1) $$A_{i}\left(\lambda \right)=\int \frac{2\pi c_{0}\cdot \varepsilon_{i}^{\prime \prime }\left(\lambda \right)}{\lambda }\cdot \left({\left|E_{i,x}\left(\lambda \right)\right|}^{2}+{\left|E_{i,y}\left(\lambda \right)\right|}^{2}+\eta {\left|E_{i,z}\left(\lambda \right)\right|}^{2}\right)dV_{i}$$

Figure 4g,h show $A_{i}\left(\lambda \right)/\left(2{\pi c}_{0}\varepsilon_{i}^{\prime \prime }\left(\lambda \right)/\lambda \right)$ to clearly see the change of total electric field intensity in the different layers of DWELL and MHA-DWELL due to the presence of MHA layer. The volume integral of electric field intensity over the whole active region of DWELL (Figure 4g) is likely to be independent of the wavelength, however the first- and second-order SPRs are not only found in the active layer of MHA-DWELL but also observed in other layers constituting MHA-DWELL, as shown in Figure 4h. The calculated absorption spectrum is used to evaluate the spectral DWELL (MHA-DWELL) response *R* (*λ*) ≈ *α* · *A*_act_ (*λ*). Here, *α* = (*e*^−^ · *λ*)/(*h* ∙ *c*_0_), where *e*^−^ is the electric charge, *h* is the Planck constant, and *A*_act_ is the total absorption in the active layer of DWELL (MHA-DWELL), as seen in Figure 4j,k. For simplicity in the calculation, we assumed that an electron-hole pair is collected for each photon absorbed in the active layer.

## 5. Electro-Optical Characterization of Plasmonic Layered Bias-Tunable DWELL Pixel Detector 

**Figure 5:**
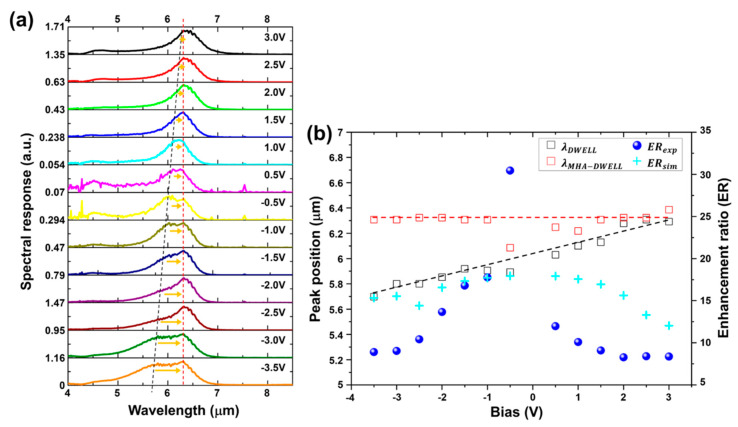
(**a**) Experimental spectral responses of MHA-DWELL pixel detector with applying the bias voltage from -3.5 V to +3.0 V at 77 K. Red (black) dash line indicates the peak position of MHA-DWELL (DWELL) response; (**b**) measured response-peak wavelengths for MHA-DWELL (red, hollow square) and DWELL (black, hollow square), and the ratio of peak response of MHA-DWELL to DWELL (measurement: blue sphere; simulation: cyan cross).

Figure 5a exhibits the measured spectral responses of the MHA-DWELL pixel detector by applying the bias between −3.5 V and +3.0 V with a step of 0.5 V at a temperature of 77 K. We can see clearly the bias tunability of the DWELL response peak wavelength, i.e., the peak wavelength of the measured spectral response of DWELL pixel detector (black dash-line) is gradually shifted toward longer wavelengths with increasing the bias from −3.5 V to +3.0 V, which results from enhanced quantum-confined Stark effect in the asymmetric DWELL structure. On the other hand, the response peaks of the MHA-DWELL pixel detector (red dash-line) are found at the wavelength of ~6.4 µm despite changing the bias, which is attributed to improving the absorption in the active layer at the SPR wavelength, as seen in Figure 4j,k. In more detail, this improved absorption in the active layer is due to the enhanced *z* component electric field caused by the presence of MHA layer in the MHA-DWELL pixel detector. Figure 4 shows that the response intensity of DWELL pixel detector at a bias of +3.0 V can be resultingly increased. However, the enhanced MHA-DWELL response was also measured at a bias of 3.0 V, and thus, we consider the effect of change in the bias, i.e., in case of the spacing between the SPR wavelength of the designed MHA and the peak wavelength of DWELL at the different bias. Note that MHA was designed to locate the MHA-DWELL response peak close to the DWELL response peak at +3.0 V as explained in the previous section, “Fabricated plasmonic layer on DWELL pixel detector”. Figure 5b shows the measured response peak wavelengths for MHA-DWELL (red hollow-square) and DWELL pixel detectors (black hollow-square), and the ratio of peak response of MHA-DWELL to DWELL at the first-order SPR wavelength (~6.4 µm). The overall agreement between experimental results and the simulated enhancement ratios at the first SPR wavelength in the bias-range from −3.0 V to +3.5 V is apparent from Figure 5b. We find that the enhancement ratios (calculated using the measured detector responses) are obtained with ~8.3 (−3.5 V), ~9 (−3.0 V), ~10 (−2.5 V), ~13.7 (−2.0 V) ~16.8 V (−1.5 V), ~17.8 (−1 V), ~30.4 (−0.5 V), ~12 (+0.5 V), ~10.1 (+1 V), ~9.1 (+1.5 V), ~8.3 (+2.0 V), ~8.4 (+2.5 V), and ~8.4 (+3.0 V). Discrepancies of response-peak wavelength and enhancement ratio in the range of small bias are probably due to the low intensity of detector response and the alignment in the electro-optical measurement. Figure 5a,b clearly demonstrate that the integration of MHA layer onto the DWELL pixel detector (MHA-DWELL) can significantly improve the DWELL sensitivity and realize the wavelength-sensitive IR detector with a narrower bandwidth, as compared to a bare DWELL detector. 

## 6. Conclusions

The results outlined here illustrate the potential of plasmonic resonance in a metal layer perforated with a two-dimensional subwavelength hole array for improving the infrared (IR) detector sensitivity and modifying the detector spectral response as desired in order to fully benefit from algorithmic spectrometry that is at the heart of next generation spectral IR sensor, just as the human eye does. Uniquely, we have demonstrated numerically and experimentally the capability for the control over the shape of bias-tunable InAs/InGaAs quantum-dots-in-a-well (DWELL) detector responses as well as the detector responsivity enhancement by integrating a fixed plasmonic structure. The measured response peaks of the plasmonic DWELL detector are found at the plasmonic resonance wavelength of ~6.4 µm, while the peak position of the DWELL detector response is shifted continuously from ~5.70 µm to ~6.30 µm with increasing the bias voltage from -3.5 V to +3.0 V. In addition, the ratio of peak response of plasmonic DWELL to DWELL at the plasmonic wavelength of ~6.4 µm is obtained in a range from ~8.3 to ~30.4. The spectral-shaping and photoresponse enhancement are achieved by the anisotropic dielectric constants of DWELL absorber and the electric field in the direction along the growth direction due to the excitation of surface plasmon resonance. The comprehensive set of data gained from computational and theoretical studies provides an insight into fundamental principles and design trade-offs of plasmonic architectures for realizing an efficient and easy-to-modify DWELL (or QD)-based IR detector.
